# Lensless Multispectral Camera Based on a Coded Aperture Array

**DOI:** 10.3390/s21227757

**Published:** 2021-11-22

**Authors:** Jianwei Wang, Yan Zhao

**Affiliations:** 1 School of Instrumentation and Optoelectronic Engineering, Beihang University, Beijing 100191, China; zhaoyan@buaa.edu.cn; 2 Aerospace Information Research Institute, Chinese Academy of Sciences, Beijing 100094, China

**Keywords:** multispectral imaging, lensless imaging, code aperture

## Abstract

Multispectral imaging can be applied to water quality monitoring, medical diagnosis, and other applications, but the principle of multispectral imaging is different from the principle of hyper-spectral imaging. Multispectral imaging is generally achieved through filters, so multiple photos are required to obtain spectral information. Using multiple detectors to take pictures at the same time increases the complexity and cost of the system. This paper proposes a simple multispectral camera based on lensless imaging, which does not require multiple lenses. The core of the system is the multispectral coding aperture. The coding aperture is divided into different regions and each region transmits the light of one wavelength, such that the spectral information of the target can be coded. By solving the inverse problem of sparse constraints, the multispectral information of the target is inverted. Herein, we analyzed the characteristics of this multispectral camera and developed a principle prototype to obtain experimental results.

## 1. Introduction

A spectrum is a type of information characterized by light in the wavelength dimension. Each substance has a different reflectivity and absorption rate for different wavelengths of light. Therefore, the spectrum reflected by the target carries the attribute information of the target, which is very beneficial for the identification and analysis of the target [1,2,3]. Multispectral imaging technology is an imaging technology that can acquire multiple band image items of information of a target. Compared with monochrome images and color images, the information obtained by this technology is richer [4,5]. Multispectral imaging technology is an imaging technology with low spectral resolution. Compared with hyperspectral imaging technology, such as in the visible and near-infrared bands, its spectral resolution is about 30 nm~50 nm, while the spectral resolution of hyperspectral imaging is usually less than 10 nm. Therefore, multispectral imaging technology can obtain about 10 bands in the visible and near-infrared bands [6,7]. In practice, the number of bands will be designed according to specific needs. This technology can be used in agriculture, forestry, hydrology, environmental monitoring, etc., and has a wide range of applications [8,9,10,11,12,13,14,15,16]. Traditional multispectral cameras can be divided into multi-camera type, filter wheel type, filter array, tunable filter type, prism spectroscopic type, and light field multispectral type based on the principle of multispectral image acquisition [17,18,19,20,21]. The multi-camera multispectral camera contains multiple imaging units. Each imaging unit acquires an image of one waveband. After the images of the different wavebands are registered by a registration algorithm, a multispectral image of the target is obtained; in the filter array-type multispectral camera, the filters of different wavelength bands are arranged in a certain direction in order, and the image of the target is passed through different filters in turn by pushing and sweeping, so as to obtain different wavelength information of the same target. A tuning filter multispectral camera uses a filter device with adjustable bands to realize imaging of different bands, while the prism split-light multispectral camera needs to place a slit on the primary image plane of the telescope and pass the prism through the slit. The target light is dispersed into several spectral channels, which are recorded by the detector, and only the spectral and one-dimensional spatial information can be obtained with a single exposure. The light-field multispectral camera uses the principle of light-field imaging and sets multiple filters at the sub-aperture so that the light field camera can obtain spectrum and spatial information at the same time. Traditional multispectral imaging methods require a complex optical and mechanical structure. The lens is limited by the size of the lens and the detector. The increase in the band causes the volume to increase, and the volume has a limit which cannot be further optimized. This theory is based on coded aperture lensless imaging technology [22,23,24,25,26,27,28] and the application of algorithms such as compressed sensing in code aperture imaging and lensless imaging [29,30,31,32], which has a strong noise suppression ability under certain conditions. In particular, Refs. [24,26] have given us a lot of inspiration, but the process cost of implementing a filter on the detector is relatively high. This paper proposes a lensless multispectral imaging system. The system is mainly composed of a coded aperture and filters. The coded aperture is composed of multiple sub-coded apertures, and each sub-coded aperture corresponds to a wavelength filter. There is no need to implement on-sensor filters through special processes, which can reduce the difficulty and cost of production. For the light sheet, the size and spacing of the sub-encoding aperture are set according to the imaging parameters and the size of the target surface of the detector, and the wavelength selection is determined by the filter. The use of the coded aperture makes the system a lensless imaging system, which is no longer limited to the size of the lens, and the volume is mainly limited by the size of the detector and the focal length of the system. Compared with a traditional multispectral imaging system, our proposed system is not only small in size and low in cost, but can also be flexibly designed according to the sparse characteristics of the target, adjust the space–spectrum aliasing, realize the compression of multispectral data, and make full use of the limited the number of pixels that the detector can record for the multispectral image information of the target. In this article, the principle of the coded aperture multispectral imaging system is introduced in detail, and then the imaging performance is evaluated through the simulation analysis method. Based on this, the principal prototype is designed, tested, and verified.

## 2. Materials and Methods

### 2.1. Principle

The design of the coded aperture multispectral camera is mainly inspired by lensless, three-dimensional (3D) imaging. The characteristics of the coded template are used to realize the recording of 3D data, and the 3D data of the target can be reversed through the algorithm. The filter array is used to compress the spatial information and spectral information of the target into a two-dimensional spatial distribution of light intensity so that spectral images can be acquired.

**Figure 1:**
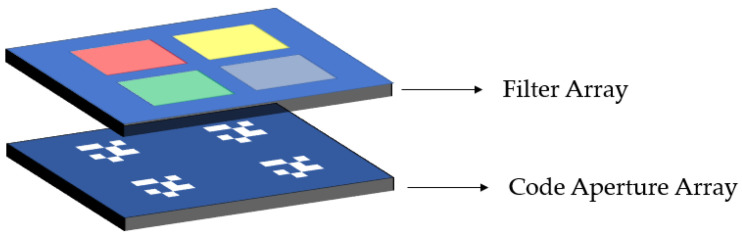
Multispectral coding aperture, including filter array and coded aperture array.

The coded aperture multispectral camera is mainly composed of a detector and a multispectral coded aperture. The multispectral coded aperture is the main imaging device. The principle is shown in Figure 1.

The multispectral coded aperture consists of two layers. The first layer is a filter array for light splitting. The number of filters is related to the number of bands. If the size is relatively large, multiple filters can be used for splicing; if the size is small, we can use coating technology to coat filter films of different wavelengths on the same glass substrate; the second layer is a coded aperture array, which is composed of several sub-coded apertures; the sub-coded apertures correspond to the filters in the filter array one-to-one, and each waveband can form a unique point spread function.

The multispectral coding aperture can be regarded as a substrate integrated with several coding apertures. Each coded aperture can independently realize the imaging function. With the corresponding filter, single-band imaging can be realized. When the coded aperture is being designed, it is divided into the following two situations.

**Figure 2:**
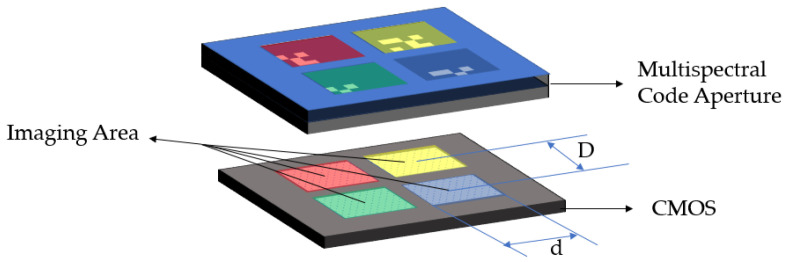
Schematic diagram of the first type of coded aperture multispectral imaging principle. The distance between the centers of the sub-encoding apertures is D, and the width of the area where the target is projected onto the detector through each sub-aperture is d. When d is less than D, the light rays passing through the sub-apertures do not interfere with each other.

The first is that the distance between the sub-coded apertures is larger than the imaging area. As shown in Figure 2, D is greater than d. At this time, the imaging areas between different sub-coded apertures do not interfere with each other, and the imaging areas are independent of each other. The coded aperture is regarded as a single coded aperture imaging system. The photosensitive surface of the detector is divided into several areas according to the wavebands, and the image is reconstructed separately, and the distance between the images of different wavebands is calibrated. The reconstructed images can be formed by a completely multispectral image. Since each band is independent, the sub-coded aperture can be designed in the same form.

**Figure 3:**
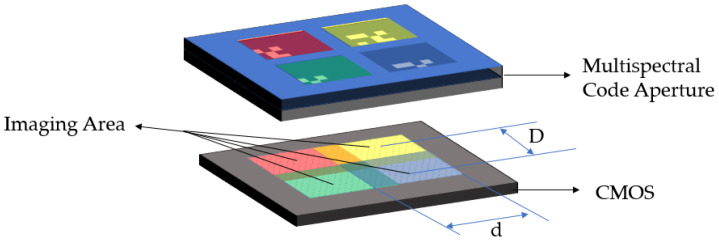
Schematic diagram of the second type of coded aperture multispectral imaging principle. When d is greater than D, the parts of light passing through different sub-apertures overlap on the detector, and the information between different wavebands is aliased.

As shown in Figure 3, the second situation is that the distance between the sub-coded apertures is smaller than the imaging area, and the light projected by the target onto the detector through different sub-coded apertures overlaps, and the information of different bands cannot be directly segmented, so each band cannot reconstruct the image of the target separately. The multispectral image of the target needs to be reconstructed using the global image recorded by the detector and the corresponding PSF. However, this creates new problems. If the coding apertures of different bands are the same, it is obvious that the PSFs of different bands are very similar. In the reconstruction result, the image of each band will have the information of other bands, which will cause errors in the reconstruction result. However, the advantage of this is that the photosensitive surface of the detector can be fully utilized, and the number of spatial pixels can be increased as much as possible under the same number of bands.

### 2.2. Reconstruction Method

Similar to the imaging optical system, the pattern received by the detector can be expressed as the convolution of the point spread function and the target. If the point spread function is expressed as h(x,y,λ), the luminous intensity of the target is expressed as L(x,y,λ) and the energy distribution received by the detector is shown in Equation (1):

(1) $$I\left(x,y\right)=\sum_{\lambda_{1}}^{\lambda_{n}}h\left(x,y,\lambda \right)⨂L\left(x,y,\lambda \right)$$

where ⨂ represents the convolution operation. If h(x,y,λ) are not correlated with each other, or their mutual convolution is close to 0, then $h\left(x,y,\lambda_{n}\right)$ can be calculated on both sides of Equation (1). We then perform correlation or convolution operations to obtain the corresponding band image $L\left(x,y,\lambda_{n}\right)$, as shown in Equation (2).

(2) $$h\left(x,y,\lambda_{m}\right)⨂I\left(x,y\right)=h\left(x,y,\lambda_{m}\right)⨂\sum_{\lambda_{1}}^{\lambda_{n}}h\left(x,y,\lambda \right)⨂\;L\left(x,y,\lambda \right)=\rho_{\lambda_{m}}L\left(x,y,\lambda \right)$$

(3) $$\rho_{\lambda_{m}}=h\left(x,y,\lambda_{m}\right)⨂h\left(x,y,\lambda_{m}\right)$$

Due to the influence of noise and errors, in practical applications, we cannot achieve good results by using convolution operations. In fact, the forward model conforms to the framework of compressed sensing, and compressed sensing algorithms can be used to reconstruct multispectral images and learn from the method in the literature [24], using *l*_1_ norm minimization, and the 3D total variation (3DTV) prior model on the scene and the low-rank prior model on the spectrum as Equation (4).

(4) $$\varepsilon =argmin\frac{1}{2}{\left\|b-Av\right\|}_{2}^{2}+\alpha {\left\|\nabla_{xy\lambda }v\right\|}_{1}+\beta {\left\|v\right\|}_{*},\;v\geq 0$$

The inverse problem can use the fast iterative shrinkage-thresholding algorithm with weighted anisotropic 3DTV.

The authors of Ref. [26] provide usable codes and usage methods. Their study mainly uses a diffuser to encode and compress the target information into a two-dimensional plane and realize 3D imaging through reconstruction. The 3D information of the target obtained is consistent with the mathematical problems involved in Ref. [26]. The code inputs are *A* and *b*, so the codes they provide can be used directly to reconstruct the image, which only needs to obtain the point spread function and the data recorded by the detector and adjust *α* and *β* according to the sparse characteristics of the scene.

### 2.3. Camera Design

To verify the feasibility and imaging performance of the proposed coded aperture lensless multispectral imaging method, a simulation method is used for analysis. Because there is essentially no difference between the first type of coded aperture multispectral imaging and panchromatic coded aperture lensless imaging, in the simulation analysis in this section, different bands are set to different sub-coded apertures, and the sub-coded apertures are randomly coded to ensure that they are not related to each other as much as possible.

**Figure 4:**
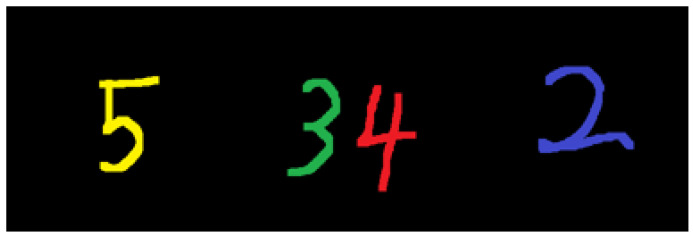
Handwritten digital simulation image. The RGB images of the three color maps can be arranged in order to simulate different band information of the target.

To facilitate the intuitive display of the accuracy of the reconstructed spectrum, the simulation target uses handwritten numbers of different colors to form a multispectral image, as shown in Figure 4.

**Figure 5:**
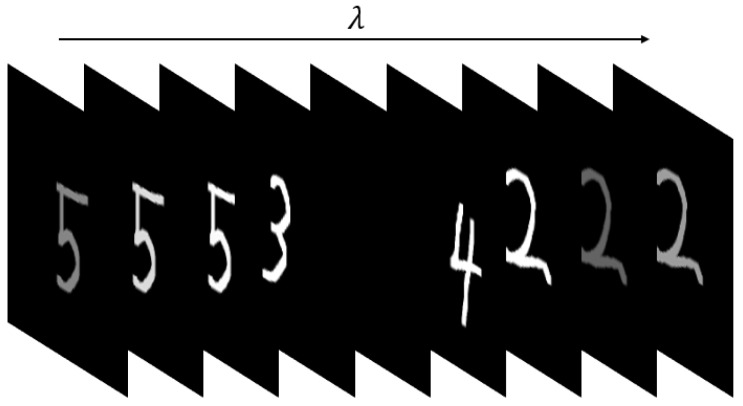
Multispectral image data of band simulation target. The RGB image of the color image of the first number 5 corresponds to the first to third bands, the RGB image of color 3 and 4 of the second image corresponds to the 4th to 6th bands, and so on.

Each color map is composed of three layers of monochromatic images, and the three-color maps are sequentially composed of a 128 × 128 × 9 multispectral target, forming the original data of the multispectral image shown in Figure 5.

**Figure 6:**
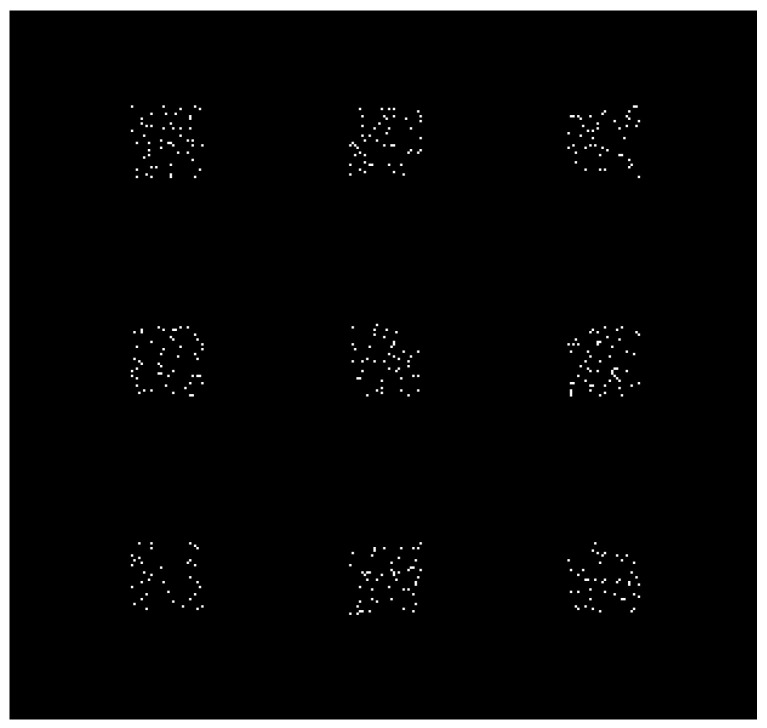
A set of 3 × 3 multispectral coding apertures. It is composed of nine different sub-encoding apertures, and each sub-aperture only encodes the image of one waveband in Figure 5, simulating imaging of different wavebands.

**Figure 7:**
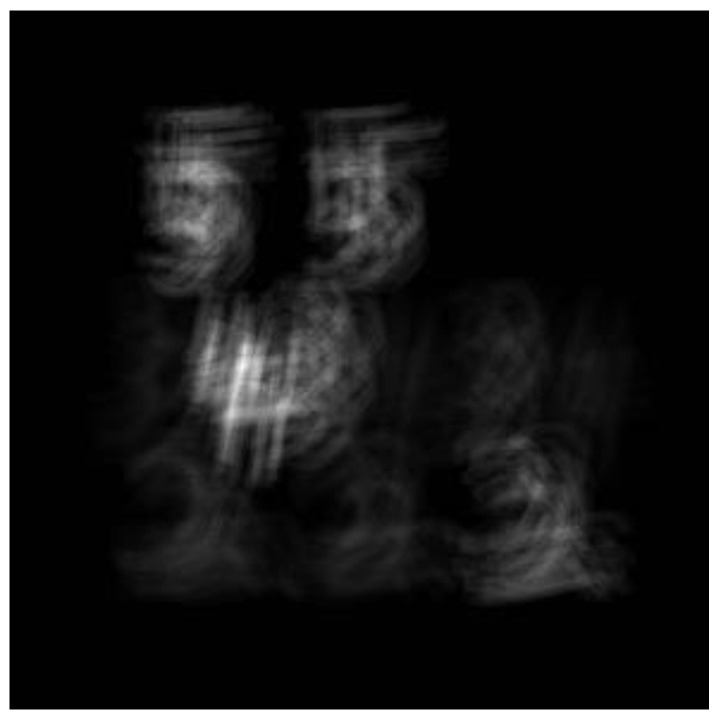
The detector receives the image which is blurred after being encoded by sub-aperture.

According to the number of bands of the simulation target, different random 3 × 3 coded apertures are used to form a multispectral coded aperture, as shown in Figure 6. The size of the aperture of the coded aperture is the same as the pixel size of the detector. There is no spectral overlap between the bands. Each band image is individually imaged through the corresponding sub-encoding aperture in the encoding aperture and finally superimposed together to form the data recorded by the detector, as shown in Figure 7.

We combined the images of the corresponding bands in the reconstruction result into a color image and calculated the Peak Signal to Noise Ratio (PSNR) to evaluate the reconstruction accuracy. By calculating the PSNR under different parameter conditions (such as D, d, and number of holes), the relationship between the PSNR and these parameters can be analyzed so that it can be referred to when designing the camera.

**Figure 8:**
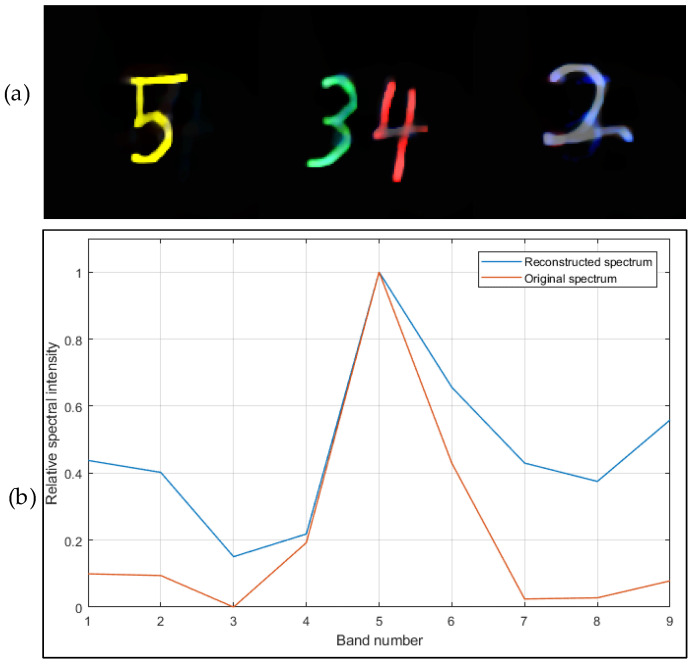
Reconstruction of the multispectral image (PSNR = 19.704); comparison of combined pseudo-color images of different bands with original data. (**a**) Color map of reconstructed image; (**b**) reconstructed spectrum of pixel *x* = 52, *y* = 45 (D = 120 px, d = 20 px, 200 iterations, constraint term coefficient is 0.01).

The reconstructed image has high accuracy and has a good effect on noise suppression. The reconstruction results are shown in Figure 8.

Since the image mostly has values 0, the value of the reconstruction result PSNR under different parameters barely changes, so root mean square error is used instead of PSNR. The smaller the root mean square error, the better the quality of the reconstructed image. Every three bands are recombined into a color image with the same color as the original image.

**Figure 9:**
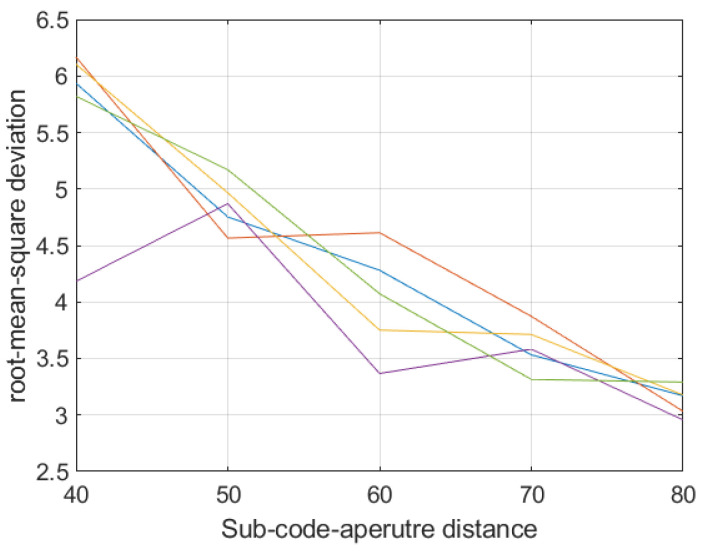
The changes in the reconstructed image quality when changing the duty cycle (proportion of small holes in coded aperture) and D of the sub-encoding aperture (sub-aperture size 20 × 20). The reconstructed image quality only shows a correlation with D (Abscissa), and there is no significant difference between different curves (each curve is calculated using different duty cycle parameters—0.1, 0.2, 0.3, 0.4, 0.5).

To obtain the optimal multispectral coding aperture parameters, the distance between the sub-coding apertures and the duty cycle of the sub-coding apertures are changed, the root mean square error of the reconstruction results is compared, and the results are shown in Figure 9.

The color of the curve in Figure 9 represents a different duty cycle. It can be seen from the figure that the duty ratio of the sub-aperture has little effect on the reconstruction result, and the difference between the reconstruction results of different duty cycles is not large. The spacing between the apertures has a greater impact on the reconstruction results, and the root mean square error is inversely proportional to the spacing; that is, the larger the spacing, the better the quality of the reconstructed multispectral image.

The smaller the distance between the sub-apertures, the more serious the aliasing between the bands, which leads to the inverse problems faced by the reconstruction method gradually developing to be ill-conditioned, which leads to the deterioration of the reconstruction results, but at the same time, the advantage is that the coding aperture is smaller. The required detector area is also smaller.

**Figure 10:**
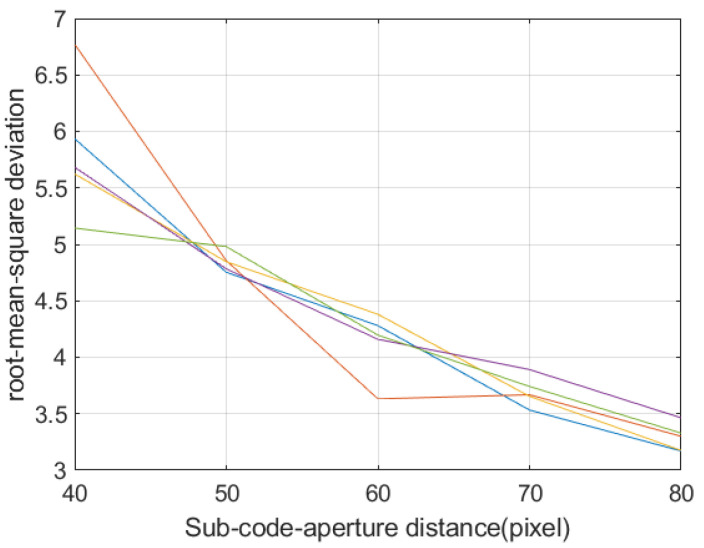
Different sub-encoding aperture size, the root mean square error of reconstructed image under different spacing conditions. (Each curve is calculated using different sizes of sub-coded aperture—20, 25, 30, 35, 40).

Figure 10 is the curve of the root mean square error of the reconstructed image with the size and spacing of the sub-encoding aperture. The size of the sub-encoding aperture has a small effect on the error of the reconstructed image. Combining the three factors, the factor that has a greater impact on the reconstructed image is the distance of the sub-encoding aperture. Therefore, if the imaging system requires high imaging quality, it needs to be as wide as possible, and a larger area detector is required. The spacing between the large sub-coded apertures reduces the aliasing between bands.

## 3. Experiment Results

### 3.1. Prototype

According to the simulation experiment in Section 2.3, when the lights passing through the sub-encoding apertures do not interfere with each other, the reconstruction result is the best. Therefore, in the experiment, the size of the target is limited to a certain size to ensure that the light passing through the sub-aperture is separated. In this case, the sub-aperture can use the same coding, and, compared to random coding, using Separable Doubly-Toeplitz Aperture (SDTA) has better reconstructed image quality. Therefore, SDTA is the better choice for making multispectral cameras. The SDTA is fixed, and nine uncorrelated SDTAs cannot be generated. Therefore, the SDTA is not analyzed in the simulation experiment.

Based on this, a prototype of a multispectral coded aperture lensless imaging system was developed. It can realize nine-band multispectral imaging, which is composed of a narrow-band filter and an array of coded apertures to form a multispectral coded aperture. Because the front-illuminated detector pixels receive light at a small angle, the energy attenuation of ±20° is usually very large. Additionally, the coded aperture is very close to the detector. If a front-illuminated detector is used, the number of pixels that receive enough signals in the final image will be very small. In the end, a back-illuminated detector of Gpixel (Chang Chun, China) was adopted: the model is GSENSE400BSI, the resolution is 2048 × 2048 px, the pixel size is 11 µm, and it has very low readout noise and dark current.

The sub-aperture adopts 31 × 31 SDTA, and the size of each light-through hole is 11 µm, consistent with the pixel size of the detector. The size of the small hole does not need to be the same as the size of the detector pixel. The size of the detector pixel can be larger or smaller, but a larger size will result in a decrease in resolution, and a smaller size will not necessarily increase the resolution.

In order to maximize the imaging quality and increase the distance between the sub-apertures, it is necessary to make full use of the photosensitive area of the detector. The size of the photosensitive area of the detector is 22.5 mm × 22.5 mm, which is divided evenly into nine areas. The final design distance between the sub-coded apertures is 7.4 mm and, finally, an image with more than 600 × 600 spatial pixels can be obtained.

The distance between the protective glass of the detector and the photosensitive surface is 0.615 mm, the thickness of the glass is 1 mm, and the thickness of the coding aperture substrate glass is 2.7 mm, so the distance between the coding aperture and the photosensitive surface is 4.315 mm.

**Figure 11:**
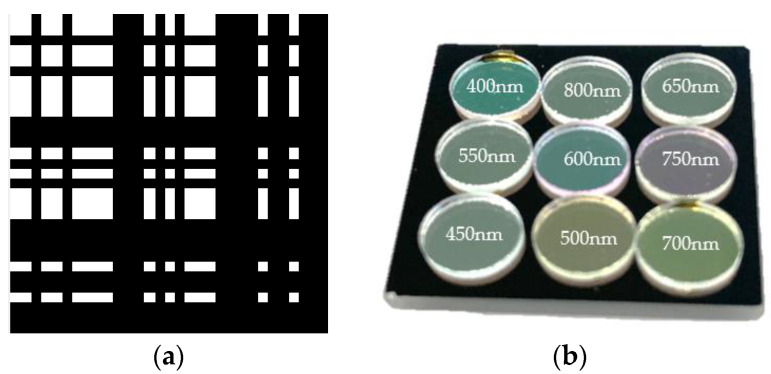
(**a**) is a designed 31 × 31 SDTA as sub-coded aperture, (**b**) is multispectral coded aperture, which consists of nine sub-encoding apertures and nine filters; each filter covers a sub-encoding aperture, and the interval between adjacent sub-encoding apertures is 7.4 mm.

The specific composition of the multispectral coding aperture is shown in Figure 11.

**Figure 12:**
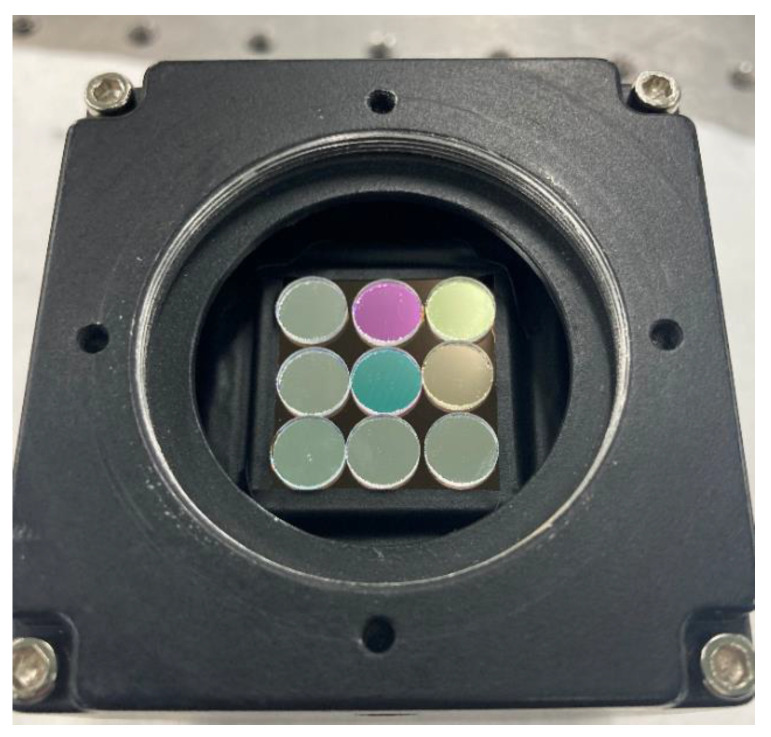
Multispectral coding aperture lensless imaging prototype.

As shown in Figure 12, the multispectral encoding aperture is coupled with the protective glass of the detector chip to make the distance between the encoding aperture and the photosensitive plane as close as possible to ensure that different bands will not be aliased.

### 3.2. Imaging Result

**Figure 13:**
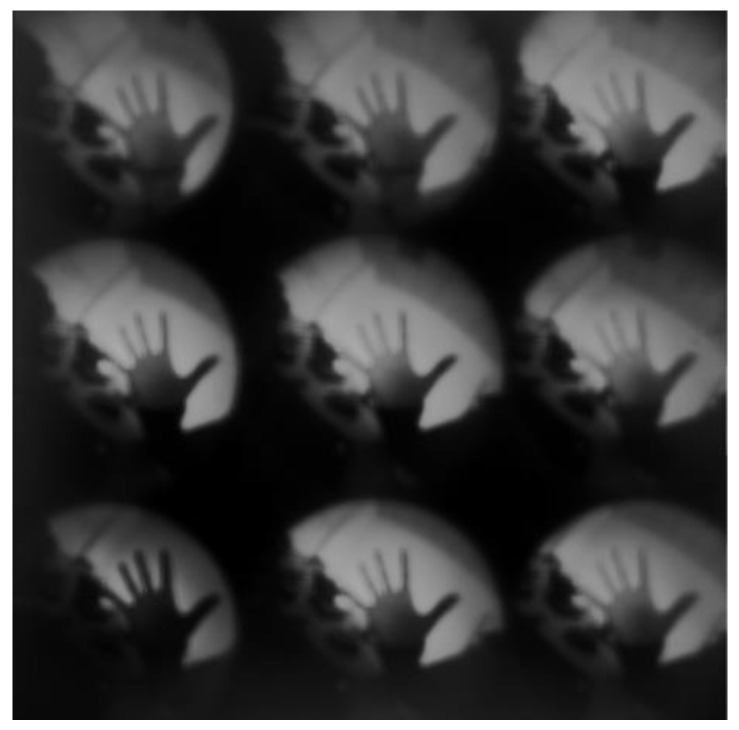
Multispectral coded aperture imaging system prototype acquisition data.

Since the sub-encoding aperture is about 0.3 mm, it also has the function of small-hole imaging, but the imaging is blurred, as shown in Figure 13.

**Figure 14:**
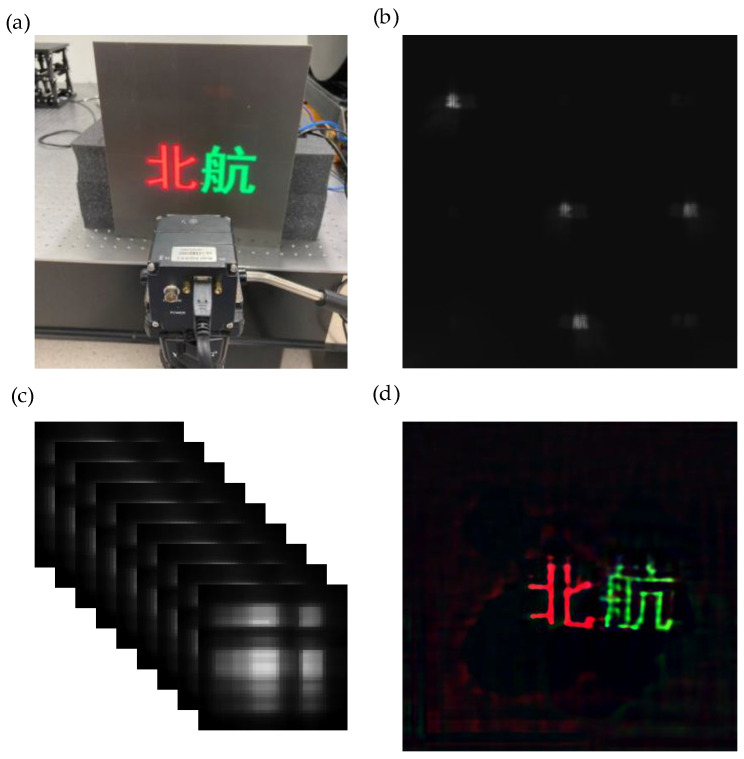
(**a**) Image of the color led screen. The camera is 28.5 mm away from the screen. (**b**) Raw data collected. Since the LED has no near-infrared light, there is no image in the near-infrared band. (**c**) Image of a single lit LED, and the obtained image is preprocessed into a nine-band PSF. (**d**) Selection of the three bands (RGB) of the reconstructed multispectral image to form a color image, which is more consistent with the color displayed on the LED screen.

An important function of the multispectral imaging system is to accurately obtain spectral information. To verify the ability of the multispectral imaging system to obtain multispectral data, the color pattern is projected on the LED display. After the data are collected, R (650 nm), G (550 nm), and B (450 nm) three-band image composite color images are obtained; if the color is consistent with the original image, it is indicated that relatively accurate spectral data can be obtained. The test results are shown in Figure 14.

The test results can better reconstruct the text displayed on the LED screen, and the color of the synthesized color image is accurate, but ghost images can be found. The main reason is that the base glass of the coded aperture of lithography is not coated with the antireflection coating, which leads to the coded hole. Part of the light is reflected by the surface of the glass in contact with the air, and then reflected by the chrome film to form a ghost image.

## 4. Discussion

Our work proposes a new multispectral imaging mode which draws on 3D lensless imaging technology, uses filters to divide the coded aperture into different regions, and uses the 3D information acquisition capability of lensless imaging to make lensless cameras able to acquire spectral information. The filter can cause different wavelengths of light to enter the lensless camera to produce different point spread functions. Through reasonable optimization design, the correlation of point spread functions between different wavelength bands can be reduced, thereby improving the reconstruction of spectral information accuracy. First, using the method of simulation analysis and randomly coded apertures, the reconstruction accuracy under different parameter conditions (D, d, and duty cycle) is analyzed. Among them, D is the most influential. The larger the D, the better the reconstruction result. The reconstructed image with complete separation between the various bands is optimal, but at the same time it needs to occupy more pixels, which requires a larger area array detector, and the volume and power consumption must be increased. It needs to be based on the sparse characteristics of the actual observation target to set an appropriate D. Finally, a principal prototype was constructed, which achieved nine-band spectral image acquisition, and imaged the LED display. The RGB three-bands were selected to synthesize color images, and good results were obtained.

Compared to fabricating filters on each pixel of the detector, we can achieve multispectral imaging by using filters that are easier to obtain. At the same time, the compactness of the imaging system can be ensured—only a multispectral encoding board is added to the sensor chip.

The imaging quality of a lensless multispectral camera has natural defects, so it cannot replace lens multispectral cameras. However, due to its compact structure, it can still play a role in specific application scenarios. For example, a chip-sized multispectral camera can be embedded in a wearable device to obtain not only color imaging, but also spectral imaging.

## Data Availability

The authors confirm that the data supporting the findings of this study are available within the article.
